# Auswirkung des COVID-19-Lockdowns auf Rettungseinsätze

**DOI:** 10.1007/s10049-021-00873-1

**Published:** 2021-04-22

**Authors:** Frank Müller, Eva Hummers, Alexandra Jablonka, Tobias Schmidt, Eva Maria Noack

**Affiliations:** 1 Institut für Allgemeinmedizin, Universitätsmedizin Göttingen, Humboldtallee 38, 37073 Göttingen, Deutschland; 2 Klinik für Rheumatologie und Immunologie, Medizinische Hochschule Hannover, Hannover, Deutschland; 3 Deutsches Zentrum für Infektionsforschung (DZIF), Braunschweig, Deutschland; 4 Malteser Hilfsdienst e. V., Diözese Hildesheim, Hildesheim, Deutschland

**Keywords:** Chronisch-Obstruktive Lungenerkrankung, SARS-CoV‑2, Notfallmedizin, Präklinische Versorgung, Notfall, Transport, Paramedic, SARS CoV‑2, Emergency medicine, Lockdown, Emergency, Transportation

## Abstract

**Hintergrund:**

Zur Bekämpfung der SARS-CoV-2-Pandemie wurden in Deutschland Mitte März 2020 weitgehende Kontaktbeschränkungen erlassen (sog. Lockdown). Die vorliegende Arbeit soll klären, welche Auswirkungen diese Maßnahmen auf Rettungsdiensteinsätze hatten.

**Material und Methoden:**

Retrospektive Auswertung von 6668 Einsatzprotokollen von vier Rettungswachen in Ostniedersachsen der beiden ersten Quartale 2020. Deskription und teststatistischer Vergleich der Einsätze sechs Wochen vor den Kontaktbeschränkungen mit einem gleich großen Zeitraum nach deren Erlass.

**Ergebnisse:**

In den sechs Wochen im Lockdown gab es 17,7 % weniger Einsätze als in den Wochen vor dem Lockdown. Insbesondere zeigte sich eine Abnahme von Einsätzen wegen Atemwegserkrankungen um 40,6 % (91 Fälle), die insbesondere auf den Rückgang von Pneumonien und exazerbierten chronisch-obstruktiven Lungenerkrankungen (COPD) zurückgeht. Gleichzeitig zeigte sich ein Anstieg des durchschnittlichen Alters der Patienten mit einer Verringerung des Anteils der unter 65-Jährigen. Veränderungen bei psychiatrischen Erkrankungen, verstorbenen oder verletzten Patienten oder der Verweigerung von Behandlung und Transport wurden nicht beobachtet. Insgesamt wurden im Zeitraum 67 Patienten (1,0 %) mit Verdacht auf oder bestätigter COVID-19-Erkrankung behandelt.

**Diskussion:**

Im Rettungsdienst zeigt sich eine Reduktion der Einsätze in Folge der Kontaktbeschränkungen, wobei diese nicht so stark ausfällt, wie für Notaufnahmen beschrieben wurde. Dieser Rückgang könnte auf eine Reduktion insbesondere weniger schwerer Erkrankungsfälle und jüngerer Patienten zurückzuführen sein. Auffällig ist die Reduktion von Pneumonien und exazerbierter COPD. Dies könnte einerseits bedeuten, dass Kontaktbeschränkungen das Infektionsgeschehen bei anderen Atemwegserkrankungen reduziert haben, aber ebenfalls, dass Patienten Krankenhausbehandlungen vermeiden wollten.

## Abstract (en)

**Background:**

As a response to the severe acute respiratory syndrome coronavirus 2 (SARS-CoV-2) pandemic, extensive contact restrictions were imposed by law in Germany as in other European countries. The present study intends to clarify the effect of these restrictions on emergency medical service (EMS) operations.

**Materials and methods:**

Retrospective chart review of EMS operation protocols over the first 6 months of 2020 (*n* = 6668 rescue missions) in four rescue stations in eastern Lower Saxony (Germany). Description and statistical comparison of operations 6 weeks before the restrictions with an equally long period after the order of the restrictions (“lockdown”).

**Results:**

During the 6 weeks after the lockdown the frequency of rescue operations decreased by 17.7%. In particular, there was a 40.6% (*n* = 91) decrease of emergency cases with respiratory diseases, mainly due to a decline of pneumonia and exacerbated chronic–obstructive pulmonary disease (COPD). At the same time, patients’ mean age increased with fewer patients under 65 years. There were no changes in the frequency of psychiatric disorders, deceased or injured patients, or refusal of treatment and transport. A total of 67 patients with suspected or confirmed SARS-CoV‑2 infection (1.0%) were observed during this period.

**Discussion:**

EMS experienced a reduction of operations as a result of contact restrictions, although not as pronounced as was recently described for emergency rooms. This supports the hypothesis that the reduction is particularly evident in less severe cases and in younger patients. The reduction in pneumonia and COPD cases is striking. On the one hand, this could indicate that contact restrictions reduce the incidence of other respiratory infections and their impact on chronic respiratory disorders, but it could also mean that patients try to avoid hospital treatment.

Die Eindämmung von COVID-19, der durch den SARS-CoV-2-Erreger verursachten Erkrankung vornehmlich der Lunge und Atemwege [1], erfolgte in Deutschland wie in vielen anderen Ländern im Frühjahr 2020 durch Maßnahmen zur Kontaktreduzierung. Diese sollten eine unkontrollierte Virusausbreitung und eine nachfolgende Überlastung der stationären Krankenhauskapazitäten verhindern [2].

## Hintergrund

In der Folge wurden Großveranstaltungen untersagt und Schulen und Universitäten für die Präsenzlehre geschlossen. Viele Unternehmen haben auf Anwesenheit ihrer Arbeitnehmer zugunsten von Tätigkeit im Homeoffice verzichtet [3, 4], Gast- und Begegnungsstätten sowie viele Geschäfte mussten initial schließen. In der Folge wurden diese Beschränkungen schrittweise wieder etwas gelockert, zunächst durch das Öffnen von Geschäften < 800 m^2^ Ladenfläche in der 17. Kalenderwoche und von allen Geschäften ab der 19. Kalenderwoche unter Beachtung entsprechender Hygiene- und Abstandsregeln (Tab. 1; [5]). Zusätzlich wurde im Verlauf die Empfehlung zum Tragen von Mund-Nasen-Bedeckungen eingeführt (sog. Community-Masken; [6]). Zusätzliche Kapazitäten zur stationären Behandlung von schwer an COVID-19 Erkrankten wurden geschaffen und elektive Behandlungen vertagt [7]. In der Folge verzeichneten Notaufnahmen in Deutschland eine deutliche Reduktion der Anzahl behandelter Patienten [8–10].

**Table 1:** 

KW	Datum	Maßnahme/Ereignis
9	29.02.2020	Erster COVID-19-Fall in Niedersachsen
11	11.03.2020	WHO ruft Pandemiefall aus
11	11.03.2020	Absage von Veranstaltungen mit über 1000 Teilnehmern
11	12.03.2020	Geplante elektive Operationen werden verschoben
11	14.03.2020	Dänemark schließt Grenze, Polen und weitere Länder folgen
12	16.03.2020	Schulen, Kindertagesstätten, Hochschulen, Museen, Theater, Einzelhandel, Kultur‑, Sport‑, Freizeit- und Vergnügungsstätten in Nds. werden geschlossen, Einschränkung von Klinik‑, Altenheim- und Pflegeheimbesuchen
12	17.03.2020	Allgemeines Tourismusverbot
12	20.03.2020	Gaststätten schließen
13	22.03.2020	Allgemeinverfügung zu Kontaktverbot tritt in Kraft
14	30.03.2020	Aufnahmestopp für Pflegeheime
17	20.04.2020	Öffnung von Einzelhandelsgeschäften < 800 m^2^ mit Hygienekonzept
18	27.04.2020	Sukzessive Wiederaufnahme des Schulunterrichts, zunächst der Jahrgänge 10–13
18	27.04.2020	Maskenpflicht („Community-Masken“) für Nahverkehr und Einzelhandel
19	11.05.2020	Gaststätten, Museen und Zoos öffnen, Kontaktverbot gelockert (2 Haushalte)
22	25.05.2020	Hotels öffnen für touristische Zwecke, Präsenzunterricht in Schulen, Fitnessstudios etc. öffnen

In der vorliegenden Arbeit werden die Häufigkeit von Rettungsdiensteinsätzen sowie die Charakteristika der behandelten Patienten vor und während der verordneten Kontaktbeschränkungen (sog. Lockdown) untersucht. Der untersuchte Zeitraum des Lockdowns umfasst dabei die Kalenderwochen 13–18, umfasst also dabei den Zeitraum, in dem Schulen, Kindertagesstätten und Einzelhandelsgeschäfte geschlossen waren und eine Allgemeinverfügung zu Kontaktverboten bestanden haben. Der Vergleichszeitraum umfasst die Kalenderwochen 7–12 vor dem Lockdown, also einen Zeitraum, in dem behördliche Anordnungen in weiten Teilen noch nicht bestanden. Dies ist gerade deshalb von Interesse, da bisherige Studien einen Rückgang von Patienten beschreiben, die sich ambulant und mit eher leichten Erkrankungsbildern vorstellen. Deren Anteil ist im Rettungsdienst mutmaßlich deutlich kleiner. Ein etwaiger Rückgang von Rettungsdiensteinsätzen und/oder eine Erhöhung des Anteils schwer erkrankter Patienten könnten dafürsprechen, dass Patienten zunächst abwarten und vermeiden, den Rettungsdienst zu rufen – auch wenn dies mit einer Verschlechterung einhergehen sollte. Zusätzlich werden in der vorliegenden Studie alle Fälle unabhängig vom Krankheitsbild inkludiert, und somit auch psychiatrische, pädiatrische oder geburtshilfliche Notfälle, die möglicherweise bei bestehenden Studien in Notaufnahmen keine Berücksichtigung finden, da keine spezifischen Behandlungskapazitäten für diese Patientengruppen in der jeweiligen Notaufnahme bzw. dem jeweiligen Krankenhaus bestehen. Die Studie soll so einen Beitrag zu einem umfassenderen Bild der Versorgungssituation liefern.

## Material und Methoden

Das Studiendesign basiert auf der DICTUM-Rescue-Studie, bei der wir Einsatzcharakteristika bei fremdsprachigen Hilfesuchenden beschreiben und eine Intervention zur besseren Verständigung entwickeln und pilotieren [11, 12]. Im Rahmen dieser Erhebung haben wir relevante Informationen aus DIVI-Protokollen (standardisiertes Notfalleinsatzprotokoll der Deutschen Interdisziplinären Vereinigung für Intensiv- und Notfallmedizin) von vier Rettungswachen in Ostniedersachsen (Wendhausen, Königslutter, Helmstedt und Braunschweig) für alle rettungsdienstlichen Einsätze (Rettungstransportwagen [RTW] und Notarzteinsatzfahrzeug [NEF]) aufgezeichnet bzw. nachprotokolliert. Das Untersuchungsgebiet ist dabei nicht streng deckungsgleich mit den Abdeckungen der Rettungsleitstellen, ergibt aber ein großes miteinander verbundenes Gebiet mit einer für Deutschland typischen Siedlungsstruktur, bestehend aus urbanen, suburbanen und ländlichen Anteilen. Bei allen Rettungswachen außer Braunschweig erfolgte die Einsatzdokumentation mit digitalem Protokollerhebungssystem („CEUS Rettungsdienst“, CKS Systeme GmbH), aus dem die Einsatzdaten exportiert und unmittelbar anonymisiert wurden. Die Dokumentation in Braunschweig erfolgte papierbasiert, die Datenextraktion wurde hier von einer erfahrenen Study Nurse und einer Hilfskraft vorgenommen.

Neben Patientenalter und -geschlecht wurden Erstdiagnosen und Glasgow Coma Scale (GCS) erhoben. Kranken- und Verlegungstransporte mittels RTW wurden in der nachfolgenden Analyse ebenso ausgeschlossen wie Fehlfahrten sowie Fahrten, bei denen kein Patient angetroffen wurde (u. a. „Bereitstellungsfahrten“ zur Unterstützung von Feuerwehreinsätzen). Während des Einsatzzeitraums ergaben sich keine substanziellen Veränderungen der Rettungsdienststruktur.

Alle statistischen Berechnungen wurden mit SPSS 26 durchgeführt, Abbildungen wurden mit GraphPad Prism 8.3 erstellt. Neben absoluten und relativen Häufigkeiten wurden Chi-Quadrat-Tests zur Testung von kategorialen Variablen und Mann-Whitney-U-Tests zur Testung von kategorialen mit metrischen Variablen verwendet. Es bestehen ein Ethikvotum der Ethikkommission der Universitätsmedizin Göttingen (9/9/18) sowie eine Übereinkunft über Datennutzung und -schutz mit den beteiligten Gebietskörperschaften sowie den Rettungsdiensten.

## Ergebnisse

**Figure 1:**
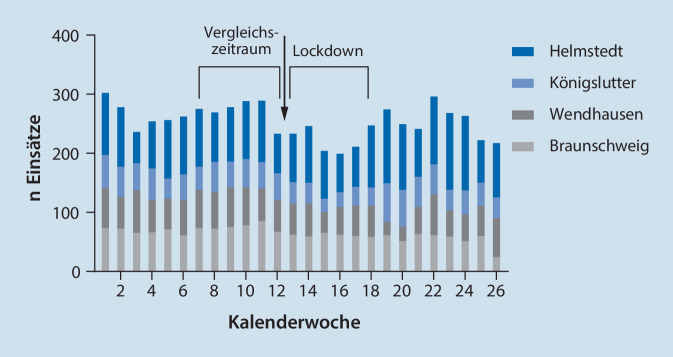

In den ersten 25 Wochen des Jahres 2020 wurden 6668 Rettungseinsätze in den beteiligten Wachen gezählt und damit im Schnitt 36,6 Einsätze (SD 7,5) pro Tag oder 256,5 Einsätze (SD 28,5) pro Woche. Die meisten Einsätze pro Woche im Untersuchungszeitraum (*n* = 305 Einsätze) fanden in der ersten Kalenderwoche statt, die wenigsten in der 16. KW (*n* = 202 Einsätze). Einsatzhäufigkeiten in Abhängigkeit von der Kalenderwoche zeigt Abb. 1. Exemplarisch wurde auch das Aufkommen der Krankentransporte im Landkreis Helmstedt geprüft, hier zeigte sich keine Veränderung der Krankentransportzahlen.

Versorgte Patienten im Untersuchungszeitraum waren im Schnitt 62,8 Jahre alt (SD 23,9 [0–104]) und bei 5,6 % (*n* = 357) der Einsätze wurden Kinder oder Jugendliche unter 18 Jahren versorgt. Das Geschlechterverhältnis der Patienten war nahezu ausgeglichen (48,9 % Frauen). Bei 245 Einsätzen (3,7 %) wurde von Patienten eine Behandlung bzw. der nachfolgende Transport ins Krankenhaus abgelehnt.

Bei einem Viertel (24,9 %) der Einsätze wurde als Verdachts- bzw. Erstdiagnose eine Herz-Kreislauf-Erkrankung dokumentiert, gefolgt von Verletzungen (20,3 %), Erkrankungen der Lungen und Atemwege (10,6 %) und neurologischen Erkrankungen (10,0 %). Der überwiegende Anteil der Patienten hatte einen initialen GCS von 15 Punkten (82,0 %). 10 oder weniger GCS-Punkte hatten lediglich 7,8 % der Patienten. Bei einem Prozent der versorgten Fälle (*n* = 67) bestand der Verdacht auf oder eine bereits bestätigte Infektion mit dem neuartigen Coronavirus SARS-CoV‑2. Bei *n* = 27 (40,3 %) der COVID-Verdachtsfälle wurde Atemwegserkrankung als Arbeitsdiagnose codiert, bei weiteren *n* = 22 (32,8 %) eine Pneumonie. Alter, Geschlecht, GCS und NACA-Score unterschieden sich nicht signifikant von Notfallpatienten ohne COVID. In den Gebietskörperschaften, in denen die beteiligten Rettungswachen liegen, stieg unterdessen die Anzahl der COVID-19-Infizierten auf bestätigte 567 Fälle [13].

### Einsatzhäufigkeiten vor und nach Lockdown

Für die weitere Auswertung wurden Einsatz- bzw. Patientencharakteristika zweier Gruppen miteinander verglichen. Die erste Gruppe spiegelt dabei alle Einsätze sechs Wochen (KW 7 bis KW 12) unmittelbar vor Inkrafttreten der Kontaktbeschränkungsmaßnahmen wider, die zweite Gruppe umfasst alle Einsätze in der Zeitspanne von sechs Wochen, in denen die Kontaktbeschränkungen bestanden. Der Beschluss zur Beschränkung sozialer Kontakte erfolgte am 22. März 2020, also am letzten Tag der KW 12.

Während im ersten Zeitraum insgesamt 1650 Einsätze verzeichnet wurden, reduzierte sich während des Lockdowns die Einsatzanzahl auf 1358, was einer Abnahme von 17,7 % entspricht. Dabei ist zu beobachten, dass ein relevanter Rückgang der Fallzahlen bereits in der Kalenderwoche 12, also unmittelbar vor dem eigentlichen Lockdown zu verzeichnen ist, wobei in dieser Woche bereits schrittweise Schulen und Einzelhandel geschlossen wurden.

**Figure 2:**
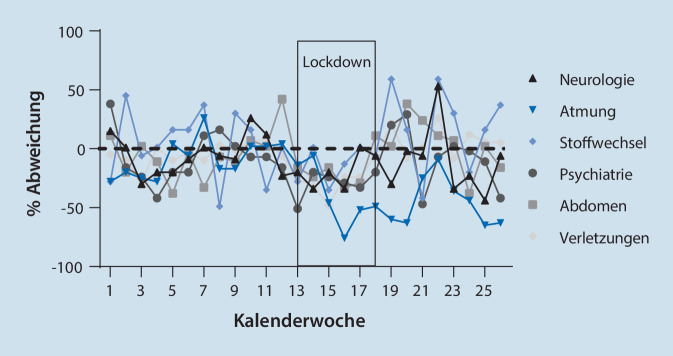

Es zeigte sich, dass das Durchschnittsalter der versorgten Patienten während des Lockdowns um etwa drei Jahre höher lag als das der versorgten Patienten vorher (*p* = 0,012). Der Anteil der über 65-Jährigen nimmt dabei zu, während der Anteil von Kindern, Jugendlichen und Erwachsenen bis 65 Jahre abnimmt. Eine Veränderung der Erkrankungsschwere (GCS und NACA-Score) lässt sich nicht feststellen. Bei den vergebenen Arbeitsdiagnosen lassen sich signifikant weniger Atemwegserkrankungen in der Periode nach Verhängung der Kontaktbeschränkungen feststellen (9,8 % vs. 13,6 %; *p* = 0,001; Tab. 2). Atemwegserkrankungen sanken im Lockdown um über 40 % ab, allerdings mit einer leichten Latenz von einer Woche (Abb. 2).

**Table 2:** 

Charakteristik		KW 7–12 (vor Lockdown)	KW 13–18 (im Lockdown)	Relative Veränderung		„Missing“
		*n* (%)	*n* (%)	(%)	*p*	*n*
Alle Patienten		1650 (100)	1358 (100)	−17,7		
**Soziodemografische Daten**						
*Geschlecht*	Männlich	811 (51,8)	650 (50,9)	−19,9	0,635	163
	Weiblich	756 (48,2)	628 (49,1)	−16,9		
*Alter (Jahre)*	Ø (SD)	61,84 (24,78)	64,28 (23,54)	+ 2,4 Jahre	**0,012**	127
	0–17	118 (7,4)	65 (5,0)	−44,9	**0,005**	127
	18–64	613 (38,6)	466 (36,1)	−24,0		
	65+	858 (54,0)	761 (58,9)	−11,3		
**Erstbefunde/rettungstechnische Aspekte**						
*GCS*	Ø (SD)	14,02 (2,82)	13,99 (2,89)	−0,03 Punkte	0,440	143
*NACA*	Ø (SD)	3,16 (1,34)	3,09 (1,40)	−0,07 Punkte	0,166	1491
*Auffälliger psychiatrischer Befund*		171 (14,3)	155 (15,6)	−9,4	0,389	823
*Behandlungsverweigerung*		54 (3,3)	46 (3,4)	−14,8	0,861	0
**Erstdiagnosen**						
*Herz und Kreislauf*		380 (23,0)	350 (25,8)	−7,9	0,081	0
*Neurologie*		172 (10,4)	140 (10,3)	−18,6	0,918	0
*Lunge und Atemwege*						0
Inkl. V. a. COVID-19		224 (13,6)	133 (9,8)	−40,6	**0,001**	
Exkl. V. a. COVID-19		215 (13,0)	105 (7,7)	−51,2	**<** **0,001**	
*Stoffwechsel*		83 (5,0)	72 (5,3)	−13,3	0,737	0
*Psychiatrie*		135 (8,2)	95 (7,0)	−29,6	0,223	0
*Abdomen*		135 (8,2)	111 (8,2)	−17,7	0,994	0
*Gynäkologie und Geburtshilfe*		17 (1,0)	16 (1,2)	−5,9	0,698	0
*Sonstige Erkrankungen*		127 (7,7)	113 (8,3)	−11,0	0,530	0
*Verletzung*	Keine	1317 (79,8)	1114 (82,0)	−15,4	0,125	0
	Leicht	211 (12,8)	158 (11,6)	−25,1	0,337	
	Mittel	101 (6,1)	74 (5,4)	−26,7	0,433	
	Schwer	21 (1,3)	12 (0,9)	−42,8	0,308	
*Exitus letalis*		51 (3,1)	47 (3,5)	−7,8	0,569	0

In der weiteren Auswertung der angegebenen Erstdiagnosen auf dem DIVI-Protokoll zeigte eine signifikante Verringerung von Fällen mit Verdacht auf Pneumonien (4,5 % auf 2,5 %; *p* = 0,004), exazerbierter COPD (3,2 % auf 1,8 %; *p* = 0,019), Orthostase bzw. orthostatischer Dysregulation (von 4,1 % auf 2,7 %; *p* = 0,038) im Untersuchungszeitraum nach den Kontaktbeschränkungen. Bei den relevanten Tracerdiagnosen Kreislaufstillstand, Schwerverletzte/Polytrauma/Schädel-Hirn-Trauma, zerebrale Ischämie und akutes Koronarsyndrom zeigten sich keine signifikanten Unterschiede zwischen den beiden untersuchten Zeiträumen.

Auch bei der durch Rettungskräfte codierten psychiatrischen Ersteinschätzung zeigten sich keine signifikanten Unterschiede, außer, dass in den Einsätzen während der Lockdownphase weniger Patienten mit depressiven Stimmungslagen auffällig wurden (Reduktion von 1,5 % auf 1,0 % der Patienten; *p* = 0,047).

## Diskussion

In unserer Untersuchung konnten wir eine Reduktion der Rettungsdiensteinsätze in den sechs Wochen nach Inkrafttreten der Kontaktbeschränkungsmaßnahmen zur Eindämmung von COVID-19 um 17,7 % im Vergleich zu den sechs Wochen vorher feststellen. Dabei zeigten sich bereits deutlich reduzierte Einsatzzahlen in der Woche vor dem Lockdown. Unsere Studie bestätigt dabei die Tendenz, die in anderen Studien im Notaufnahmesetting festgestellt wurde, bei denen eine deutliche Verringerung der Patientenanzahl verzeichnet wurde [8, 9, 14, 15]. Ein direkter Vergleich fällt dabei schwer, da diese Studien meist Rückgänge im Vergleich zum Vorjahreszeitraum berechneten. Aus den von Slagman et al. erhobenen Daten lässt sich jedoch eine Abnahme um teilweise bis zu 40 % in den Wochen des Lockdowns im Vergleich zu vorangehenden Wochen kalkulieren [8]. Die Autoren erklären die Unterschiede in Notaufnahmen vor allem durch den starken Rückgang von sich ambulant vorstellenden Patienten mit weniger dringlichen Behandlungsfällen (Triagekategorie „grün“ bzw. „blau“), deren Anteil mutmaßlich bei Rettungsdiensteinsätzen niedriger liegt. Die Reduktion der Patientenzahlen lässt sich im Rettungsdienstsetting wesentlich auf den Rückgang von Erkrankungen der Atemwege und Atmungsorgane zurückführen (insbesondere Pneumonien und exazerbierte COPD). Dies steht im Einklang mit der verminderten Vorstellung von Patienten mit COPD in deutschen Notaufnahmen, die Möckel et al. feststellten. Die Autoren vermuten, dass die Kontaktbeschränkungen die infektbedingten Exazerbationen verringert haben könnten [6]. Es muss allerdings ebenfalls beachtet werden, dass der Zeitpunkt des Lockdowns auch in einen Zeitraum fällt, in dem Grippewellen auch ohne Lockdown abebben würden, und entsprechend auch in anderen Jahren in diesem Zeitraum eine Reduktion von schweren Atemwegsinfekten zu verzeichnen wäre. Allerdings erscheint die gezeigte Halbierung der um COVID-Verdachtsfälle bereinigten Atemwegserkrankungen in der Lockdownphase bemerkenswert und kaum alleinig auf das Ende der Grippesaison zurückzuführen. Dieser Rückgang der Raten an Rettungsdiensteinsätzen bei Atemwegsinfektion ist kongruent zum abrupten Rückgang der Atemwegserkrankungen in der deutschen Bevölkerung [16].

Zudem verzeichneten wir einen Rückgang von Einsätzen, bei denen eine orthostatische Dysregulation zugrunde lag, was darauf hindeuten kann, dass bei kollaptischen Ereignissen seltener der Rettungsdienst gerufen wurde. Denkbar wäre, dass insbesondere ältere gefährdete Patienten, die ihre Alltagsmobilität einschränkten, (prä)kollaptische Ereignisse vermehrt in der Häuslichkeit hatten, wo diese eher durch eigene Hydrierung und ggf. nachfolgende Vorstellung bei Hausärzten behandelt wurden.

In unserem Setting in Ostniedersachsen konnte weder eine Erhöhung der Anzahl von Einsätzen mit Patienten mit psychiatrischen Erkrankungen noch erhöhte Raten an Todesfällen oder Ablehnung von Behandlungen festgestellt werden. Dies scheint vor dem Hintergrund relevant, dass eine Studie aus einem COVID-19-Hochprävalenzgebiet einen deutlichen Anstieg an reanimationspflichtigen Patienten und Todesfällen im Rettungsdienst berichtet [17].

Das im Rahmen dieser Studie untersuchte Versorgungsgebiet der rekrutierenden Rettungswachen, die sowohl großstädtische als auch kleinstädtische und ländliche Anteile der benannten Region versorgen, ist im Hinblick auf die Bevölkerungsstruktur prototypisch für viele andere deutsche Regionen. Durchschnittsalter, Bruttoinlandsprodukt pro Kopf und Arbeitslosenquote entsprechen denen des Durchschnitts der Bundesrepublik Deutschland. Lediglich die Bevölkerungsdichte weicht ab. Nichtsdestotrotz bedeutet die vergleichsweise geringe Anzahl an Fällen und rekrutierenden Rettungswachen in einem umschriebenen Gebiet mit etwa 341.000 Einwohnern eine Limitation unserer Studie, die eine allgemeine Generalisierung der Ergebnisse erschwert. Die gute Datenqualität sowie die Tatsache, dass keine relevanten strukturellen oder organisatorischen Veränderungen während der untersuchten Zeitspanne auftraten, sind als Stärken der Studie zu nennen. Das gewählte Studiendesign, bei dem zwei benachbarte Zeiträume innerhalb eines Jahres untersucht werden, besitzt den Nachteil, dass es für mögliche saisonale Confounder, etwa Feiertage oder Influenzawellen, nicht kontrolliert und Effekte des Lockdowns sowohl über- als auch unterschätzt werden können. So fallen in den Vor-Lockdown-Zeitraum keine gesetzlichen Feiertage, in den Lockdownzeitraum hingegen Ostern und der Maifeiertag. Andererseits können Vergleiche mit Vorjahreszeiträumen ebenfalls fehlleitend sein, etwa wenn saisonale Erkrankungen nuanciert verzögert eintreten, etwa eine Grippewelle früher abflacht oder prolongiert oder diese womöglich gerade durch die Lockdownmaßnahmen beendet wurde. Dieser Aspekt ist gerade im Frühjahr im Hinblick auf die Grippevirusverbreitung höchst relevant [16].

## Fazit für die Praxis

- Rettungsdiensteinsätze nahmen während des Lockdowns im März und April 2020 um 17,7 % im Vergleich zu den Vormonaten ab. Die versorgten Patienten waren signifikant älter.
- Am deutlichsten zeigte sich eine Reduktion von Einsätzen mit Lungen- und Atemwegserkrankten um mehr als 40 %.
- Bei Einsätzen mit psychiatrisch Erkrankten und Herzkreislauferkrankten zeigten sich keine relevanten Änderungen. Auch die Anzahl der Einsätze mit verstorbenen Patienten blieb gleich.
